# Managers' Freelancer Mindset Orientation and relational evaluations as drivers of pay decisions for external workers

**DOI:** 10.3389/fpsyg.2026.1939932

**Published:** 2026-10-16

**Authors:** Hilla Peretz, Aharon Tziner, Cristinel Vasiliu, Andreea Saseanu

**Affiliations:** 1 Braude College of Engineering Karmiel, Karmiel, Israel; 2 Tel-Hai College, Tel Hai, Israel; 3 ASE, Bucharest, Romania

**Keywords:** external workers, Freelancer Mindset Orientation, inclusion practices, pay equality, relational evaluations

## Abstract

**Purpose:**

Organizations increasingly rely on freelancers, yet pay parity between freelancers and comparable employees varies widely. This study introduces Freelancer Mindset Orientation (FMO), managers' perception of freelancing as a legitimate, attractive, and personally resonant career mode, and examines how it shapes pay equality decisions for external workers through relational evaluations and organizational inclusion practices.

**Design/methodology/approach:**

Survey data from 353 managers recruited via the Prolific platform were analyzed using path analysis in Mplus 9, with bias-corrected bootstrapped confidence intervals (5,000 resamples) to test mediation and moderation hypotheses.

**Findings:**

Managers' Freelancer Mindset Orientation (FMO) was positively associated with pay equality for freelancers and with more favorable relational evaluations of freelancers (perceived fit, commitment/loyalty, and trustworthiness). Relational evaluations were, in turn, positively associated with pay equality and partially explained by the FMO-pay association. The positive association between FMO and pay equality was stronger among managers reporting higher organizational inclusion of freelancers. Inclusion also strengthened the association between FMO and relational evaluations, whereas the association between relational evaluations and pay equality did not vary significantly across inclusion levels.

**Research limitations:**

The cross-sectional design limits causal inference. Future research should employ longitudinal designs, multi-source data (payroll records, freelancer reports), and cross-national samples.

**Practical implications:**

Organizations can promote pay fairness by developing managerial FMO through training, building trust-based manager-freelancer relationships, and implementing inclusive practices that integrate freelancers into organizational workflows.

**Originality/value:**

This study introduces FMO as a novel identity-based micro-level construct and provides the first empirical test of how managerial career orientations, relational evaluations, and inclusion practices jointly determine pay equality for external workers.

## Introduction

Organizations, more than ever, are relying on freelancers to access specialized expertise, scale capacity quickly, and remain agile under uncertainty (Barley et al., 2017; Vallas and Schor, 2020). Freelancers, self-employed external workers engaged through time-limited or ongoing contractual arrangements, are increasingly embedded in expert, knowledge-intensive, and strategically consequential work (Kozica et al., 2014). Research on digital labor platforms further indicates that freelancing often constitutes a sustained career form rather than a series of isolated gigs (Gussek and Wiesche, 2024). Yet organizations vary substantially in how they compensate freelancers. In some contexts, freelancers receive pay approximating internal benchmarks; in others, they face systematic discounting that reflects enduring categorical distinctions between “insiders” and “outsiders” (Pfeffer, 1998).

This variation matters because pay practices toward freelancers influence (i) the quality of external talent organizations can attract, (ii) the stability of boundary-spanning knowledge flows, and (iii) perceptions of organizational legitimacy (Ashford et al., 2007; Barley et al., 2017; van den Groenendaal et al., 2023). A key micro-foundation remains under-theorized: pay decisions are enacted by managers who interpret freelancing through their own cognitive and identity-based lenses rather than simply applying objective market rates or HR policies (Zadik et al., 2019; Shwartz-Asher et al., 2025).

Under the ambiguity that characterizes freelance work, managers rely on identity-relevant judgments (Who is “like us”?) and relational judgments (Who is “safe,” “committed,” and “deserving”?) (Ashforth and Mael, 1989; Hogg and Terry, 2000; Stryker and Burke, 2000). Identity theory and self-categorization perspectives suggest that individuals are more likely to extend positive evaluations and resources to those they perceive as psychologically proximate in-group members.

To address this gap, we focus on Freelancer Mindset Orientation (FMO), the extent to which managers view freelancing as a legitimate, attractive, and personally resonant career mode. We conceptualize FMO as a career-related identity orientation that captures whether managers perceive freelancing as a viable and respectable form of professional achievement or as a marginal deviation from “real” employment (Ashford et al., 2018; Vallas and Schor, 2020).

Building on career-identity and self-categorization perspectives (Ashforth and Mael, 1989; Hogg and Terry, 2000; Stryker and Burke, 2000), we conceptualize managers' orientation toward freelancing as expressed through three related but distinct facets: personal openness, category-level evaluation, and behavioral endorsement. Identity-based perspectives suggest that responses to a social or occupational category depend partly on its relevance to the self and on the value attached to that category (Ashforth and Mael, 1989; Hogg and Terry, 2000; Miscenko and Day, 2016). Applied to freelancing, personal openness captures the extent to which managers can envision freelancing as a viable career option for themselves, reflecting the psychological proximity of the freelancer category to their own career self-concept. Category-level evaluation captures managers' broader assessment of freelancing as a legitimate and desirable labor-market form. Finally, behavioral endorsement captures the extent to which this favorable orientation is expressed in managers' willingness to deploy freelancers in organizational roles. This distinction is also consistent with career-orientation research showing that individuals' views of careers involve both self-referential career preferences and behavioral orientations toward alternative career arrangements (Hall, 2004; Briscoe and Hall, 2006).

Mapping this logic onto the freelance context therefore yields three FMO components: (a) personal openness to freelancing as a career option, (b) general evaluation of freelancing as a labor-market trend, and (c) role-based preference for deploying freelancers in organizational positions. These components are theoretically related but not interchangeable. A manager may, for example, regard the growth of freelancing positively without wishing to become a freelancer personally, or may be personally open to freelancing while still preferring salaried employees for particular organizational roles. Such divergence is theoretically meaningful rather than measurement error. Accordingly, we conceptualize FMO as a formative composite of related but distinct facets, rather than as a reflective construct in which each indicator is assumed to be an interchangeable manifestation of the same underlying trait (Diamantopoulos and Winklhofer, 2001). Legitimacy is therefore treated as a matter of degree: even among managers who work with freelancers, meaningful variation may exist in the extent to which they regard freelancing as a legitimate, attractive, and organizationally valuable career form.

Existing career-orientation constructs do not adequately capture managers' evaluative stance toward freelancing as a workforce arrangement. Protean career orientation (Hall, 2004; Briscoe and Hall, 2006; Hamrick et al., 2025). and boundaryless career orientation (Arthur and Rousseau, 2001; Arthur, 2014; Sullivan and Arthur, 2006; Guan et al., 2019) describe how an individual manages their own career (self-direction, values-driven decision-making, physical/psychological mobility across organizations), but they are agnostic about whether the perceiver regards freelancing specifically as legitimate, and they were not designed to predict how a manager treats other people's pay. Similarly, entrepreneurial identity aspiration (Farmer et al., 2011; Hamrick et al., 2025) captures the construction of a possible future entrepreneurial self, but centers on venture creation rather than on freelance labor arrangements or on evaluations of third-party workers. No existing construct simultaneously captures (a) a manager's personal openness to freelancing as viable for themselves, (b) their general evaluation of the legitimacy of the freelance labor-market trend, and (c) their behavioral preference for deploying freelancers in organizational roles, combined into a single career-identity orientation that is theorized to predict third-party (freelancer) treatment. We therefore introduce Freelancer Mindset Orientation (FMO) to fill this gap. FMO is conceptually and empirically distinct from protean/boundaryless career orientation because it is other-referential in its consequences (it predicts how managers treat freelancers, not how managers manage their own careers) and category-referential in its content (attitudes toward an occupational category rather than toward one's own mobility). As elaborated above and in the Measures subsection, the three FMO components are conceptualized as related but non-interchangeable facets and are therefore combined as a formative composite rather than modeled as reflective indicators of a single latent attitude (Diamantopoulos and Winklhofer, 2001). We further propose that managers' relational evaluations of freelancers, operationalized as perceived fit, commitment/loyalty, and trustworthiness, constitute a key mechanism through which FMO is associated with pay decisions. We focus on these three facets because they capture complementary judgments about whether an external worker is a suitable and dependable organizational relationship partner. Perceived fit captures compatibility between the freelancer and the organization, particularly alignment with organizational values and norms, a central concern in person–organization fit theory (Kristof-Brown et al., 2023). Perceived commitment/loyalty captures managers' expectations that the freelancer will remain engaged and act with regard for organizational interests despite operating outside a conventional employment relationship. This judgment is particularly relevant in freelance relationships, in which mutual obligations and expectations are less fully specified by formal employment arrangements and therefore depend more heavily on perceived reciprocity and psychological-contract expectations (Rousseau, 1995; van den Groenendaal et al., 2023). Perceived trustworthiness captures managers' confidence that freelancers can be relied upon when managers must accept vulnerability despite limited ability to monitor or control their actions, consistent with organizational trust theory (Mayer et al., 1995; Rousseau et al., 1998).

Together, these facets capture three complementary relational considerations-compatibility, expected relational commitment, and confidence in the other party-that are especially salient when evaluating workers positioned outside the formal organizational boundary. Prior research on freelancers similarly identifies fit and loyalty as concerns in managers' evaluations of external workers and highlights the importance of reputation, trust, and relationship expectations in freelance employment relationships (Kokkodis and Ipeirotis, 2016; Zadik et al., 2019; van den Groenendaal et al., 2023). We therefore use fit, commitment/loyalty, and trustworthiness jointly to represent managers' relational evaluations of freelancers in the present study, as elaborated further in the Measures subsection. We theorize that inclusion practices, in contrast, operate as a contextual moderating condition that shapes the extent to which managerial orientations and evaluations are associated with pay outcomes (van den Groenendaal et al., 2023). The model assigns evaluations and inclusion to different structural roles because they operate at different levels of analysis and answer different theoretical questions. Relational evaluations are a psychological mechanism internal to the manager: they are the cognitive appraisal (fit, commitment/loyalty, trustworthiness) through which a stable identity-based orientation (FMO) is translated into a specific judgment about a specific freelancer, consistent with attitude-to-behavior process models in which stable dispositions operate on behavior partly through target-specific appraisals (Glasman and Albarracín, 2006; Petty and Cacioppo, 1986). Evaluations therefore are theorized as a mediating mechanism: target-specific appraisals through which a broader orientation toward freelancing may be associated with pay judgment (Rousseau, 1995). Inclusion, in contrast, is an organizational-level structural condition, not a manager-internal appraisal: it describes whether the organization's formal practices grant freelancers access to workflows, information, and social integration. Inclusion does not represent the proposed psychological mechanism linking FMO with pay; instead, it changes the strength of the FMO-pay association by determining whether a favorable managerial orientation can be enacted at all. In low-inclusion contexts, a manager with high FMO has few opportunities to act on that orientation because the freelancer is structurally segregated from decision-relevant information and workflows; in high-inclusion contexts, the same orientation has more behavioral room to translate into an actual pay decision. This person-situation interactionist logic (Kristof-Brown et al., 2023) is the definition of a moderator, a variable that changes the strength or direction of an existing relationship, rather than a mediator.

This study makes three contributions motivated directly by the gap identified above. First, prior research on freelancer compensation has been dominated by macro-structural accounts (market rates, institutional regulation) and has lacked a construct that captures the manager-level identity orientation shown here to predict pay decisions; we address this gap by introducing and empirically testing FMO as a micro-level, identity-based managerial orientation, conceptually distinguished from adjacent constructs precisely because it is other-referential (predicting treatment of freelancers) rather than self-referential (predicting one's own career mobility). Second, because an identity-based orientation is unlikely to translate automatically into a concrete resource-allocation decision, we theorize and test relational evaluations as the psychological mechanism through which FMO is converted into pay judgments, extending relational and psychological contract perspectives (Rousseau, 1995) with a specific identity-to-appraisal-to-outcome pathway. Third, because identity and appraisal alone may be insufficient when freelancers are structurally excluded from organizational workflows, we position organizational inclusion practices as a boundary condition that determines whether favorable orientations and evaluations can actually be enacted as pay parity, aligning inclusive-HRM research (van den Groenendaal et al., 2023) with distributive justice theory. Together, these three contributions substantiate FMO's value: it is not merely a redescription of existing career attitudes, but a theoretically distinct predictor whose consequences for a specific, consequential organizational outcome had not previously been modeled or tested.

### Freelancer mindset orientation and pay decisions

Drawing on identity theory and self-categorization perspectives (Ashforth and Mael, 1989; Hogg and Terry, 2000; Stryker and Burke, 2000), we argue that managers' career-related self-understandings shape how they interpret the legitimacy and value of freelancing. Individuals tend to evaluate work arrangements more favorably when those arrangements feel psychologically proximate and identity-consistent (e.g., Ashforth and Mael, 1989; Hogg and Terry, 2000). Managers who score higher on FMO are more likely to view freelancing as a viable, respectable, and desirable career path, rather than as a marginal substitute for “real” employment (Vallas and Schor, 2020). This orientation should reduce categorical distancing toward freelancers and weaken the impulse to justify differential treatment solely based on employment status.

Pay decisions are not purely market transactions; they are also symbolic statements about worth, legitimacy, and relative standing (Pfeffer, 1998). When managers adopt a mindset that normalizes and legitimizes freelancing, they should be less inclined to see discounted pay as natural or defensible and more inclined to support pay levels that reflect comparable contribution and expertise (Ren et al., 2023; Zadik et al., 2019).

*Hypothesis 1. Freelancer Mindset Orientation (FMO) will be positively related to pay equality*.

### FMO and relational evaluations of freelancers

While FMO may shape pay judgments directly, we propose that it also influences how managers perceive freelancers as relationship partners. Managers routinely evaluate whether external workers are “good partners”: Do they fit the organization's norms and values? Will they stay engaged and deliver reliably? Can they be trusted with information and autonomy? Such judgments are particularly salient when workers sit outside formal control systems (Kokkodis and Ipeirotis, 2016; Ren et al., 2023). Perceptions of fit, commitment, and trust are central to how managers interpret risk and deservingness in quasi-employment relationships (Rousseau, 1995).

Managers high in FMO should make more favorable relational evaluations for three reasons. First, identity proximity generates more charitable interpretations of ambiguous behavior, reducing attributional bias (Ashforth and Mael, 1989; Hogg and Terry, 2000). Second, managers who see freelancing as a legitimate career mode may interpret external workers' independence as normal rather than as low commitment. Third, FMO may reduce status-based suspicion, making it easier to infer trustworthiness and cultural fit even without employment-based signals (van den Groenendaal et al., 2023).

*Hypothesis 2. Freelancer Mindset Orientation (FMO) will be positively related to managers' relational evaluations of freelancers (fit, commitment/loyalty, and trustworthiness)*.

Although the possibility of reverse causality exists, whereby positive evaluations of freelancers could reinforce FMO over time, the directional sequence proposed here is grounded in attitude-judgment theory, which holds that stable dispositional orientations form prior to and shape episode-specific judgments of social objects (Glasman and Albarracín, 2006; Holland et al., 2002; Petty and Cacioppo, 1986). FMO, as a relatively stable career orientation, is thus conceptualized as antecedent to the contextually activated relational judgments managers make about specific freelancers.

### Relational evaluations and pay

Managers frequently justify pay decisions using relational arguments about deservingness and risk. Social exchange and relational contract perspectives hold that pay allocation signals perceived partner value: managers extend greater rewards to workers they view as reliable, trustworthy, and aligned with organizational goals (Rousseau, 1995; Pfeffer, 1998). Perceived fit signals that a worker can be embedded in ongoing workflows without extensive supervision, reducing the operational risk managers associate with external contributors (Kristof-Brown et al., 2023). Perceived commitment and loyalty indicate willingness to act with organizational interests in mind, making the worker a safer investment for high-stakes work (Stamper and Masterson, 2002; van den Groenendaal et al., 2023). Perceived trustworthiness determines whether managers are willing to share information and advocate for equitable pay, since trust reduces the psychological cost of treating an outsider as an insider (Schulz et al., 2022; Rousseau et al., 1998). By contrast, workers seen as misaligned or unreliable are more easily categorized as “transactional” vendors for whom discounting appears justifiable (Pfeffer, 1998; Zadik et al., 2019; Shwartz-Asher et al., 2025).

*Hypothesis 3. Managers' relational evaluations of freelancers, capturing perceived fit with organizational culture and values, perceived commitment/loyalty, and perceived trustworthiness, will be positively related to pay equality, such that more favorable relational evaluations predict greater managerial willingness to offer freelancers pay commensurate with comparable employees*.

### Evaluations as the mechanism linking FMO to pay

We propose a mediation pathway in which FMO shapes pay decisions not only through a direct attitudinal route but also through the relational interpretations managers construct about freelancers. The logic rests on three linked steps: managers high in FMO are less likely to impose categorical insider-outsider distinctions and more likely to evaluate freelancers favorably (H2); positive relational evaluations function as deservingness signals that justify pay parity (H3); and together, these steps imply that FMO operates on pay partly through its upstream effect on evaluations. This indirect pathway is consistent with attitude-to-behavior process models that distinguish between a direct route from stable dispositions to outcomes and an indirect route mediated by target-specific appraisals (Glasman and Albarracín, 2006; Holland et al., 2002; Petty and Cacioppo, 1986). We expect the mediation to be partial: managers with high FMO may also support pay equality on principled grounds independent of any specific freelancer's relational qualities, preserving a residual direct effect (Rousseau, 1995; Pfeffer, 1998).

*Hypothesis 4. The relationship between FMO and pay will be mediated by managers' evaluations of freelancers*.

### Inclusion as a moderating condition

Finally, we theorize that the translation of orientations and evaluations into pay is moderated by the inclusion context. Inclusion refers to the extent to which freelancers are granted access to organizational work practices and social or informational resources. Emerging work on inclusive HRM shows that organizations differ widely in whether they treat freelancers as peripheral “vendors” or as quasi-members (van den Groenendaal et al., 2023).

In high-inclusion contexts, managers have more frequent and higher-quality interaction with freelancers, making FMO more likely to be enacted in concrete compensation choices. When freelancers are included in key workflows, it becomes harder to sustain structural discounting while simultaneously relying on them for high-discretion work (van den Groenendaal et al., 2023).

*Hypothesis 5. Inclusion will moderate the relationship between FMO and pay, such that the positive relationship will be stronger when inclusion is higher*.

Similarly, when inclusion is high, positive relational evaluations are more actionable: managers can justify higher pay because freelancers are embedded in critical workflows and their reliability is visible (van den Groenendaal et al., 2023).

*Hypothesis 6. Inclusion will moderate the relationship between evaluations and pay, such that the positive relationship will be stronger when inclusion is higher*.

In addition to moderating the direct FMO-pay and evaluations-pay paths, inclusion should also condition the strength of the relationship between FMO and relational evaluations. In high-inclusion contexts, managers interact with freelancers more frequently and with higher quality (e.g., shared meetings, joint access to organizational information, collaborative workflows), (van den Groenendaal et al., 2023; Nishii and Leroy, 2022), which gives a favorable career-identity orientation (FMO) more behavioral opportunity to be enacted as positive relational judgments; concretely, a manager who views freelancing as legitimate (high FMO) has more occasions to observe a freelancer's reliability, cultural fit, and trustworthiness when that freelancer is embedded in organizational routines than when the freelancer is structurally segregated (van den Groenendaal et al., 2023). We therefore add:

*Hypothesis 7. Inclusion will moderate the relationship between FMO and managers' relational evaluations of freelancers, such that the positive relationship will be stronger when inclusion is higher*.

In sum, FMO is expected to relate to pay decisions directly (H1) and indirectly through relational evaluations (H2-H4). Inclusion is conceptualized as a contextual moderator of the FMO-pay relationship (H5), the evaluations-pay relationship (H6), and the FMO-evaluations relationship (H7). The conceptual model is presented in Figure 1.

**Figure 1:**
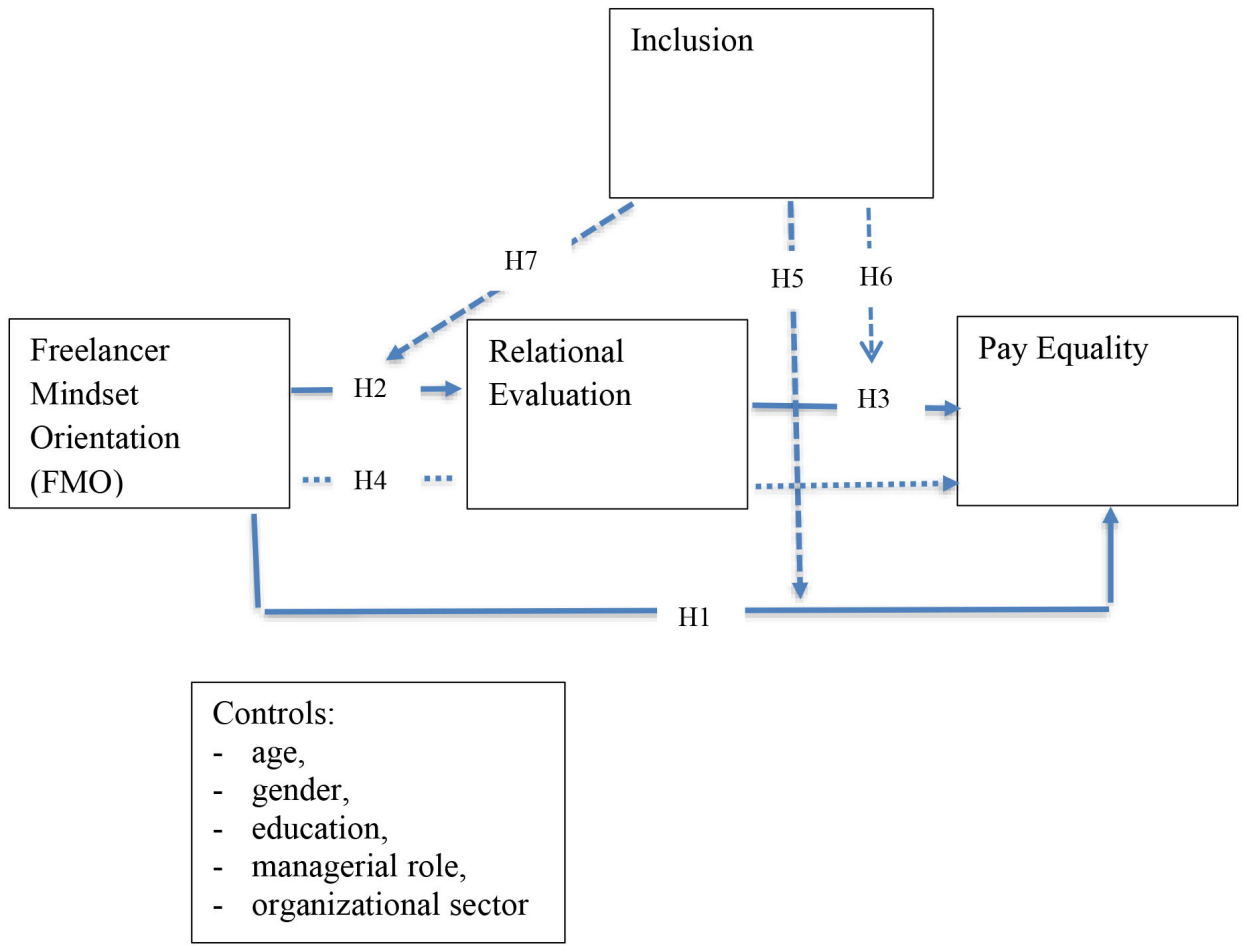
The research model. Solid arrows represent hypothesized direct relationships. Dashed arrows indicate moderation of the relationship on which they terminate. H4 represents the hypothesized indirect relationship between FMO and pay equality through relational evaluations.

## Method

### Participants and procedure

Data for this study were collected in Romania using an online platform. Participants were recruited via the Prolific online research platform and completed an anonymous, self-administered questionnaire in English. Romania was selected as the sampling context because the Prolific platform maintains a large, verified panel of Romanian managers with documented English proficiency, enabling cost-effective access to a managerial population with direct experience managing mixed workforces. Romanian managers operate in a transitional labor market context where freelance work arrangements have grown substantially alongside EU labor mobility and platform-mediated project work, making the sample highly relevant to the research question. English was used as the survey language because Prolific verifies respondents' English proficiency as part of panel registration.

Freelancers were defined as self-employed professionals working under time-limited arrangements, explicitly excluding individuals employed through staffing or manpower agencies. This exclusion was necessary to maintain conceptual precision: agency workers maintain a formal employment relationship with the intermediary agency, not the focal organization, and differ from self-employed freelancers in their psychological contract structure, degree of organizational integration, and the managerial discretion available over their compensation. Before beginning the questionnaire, respondents provided informed consent. Participants received monetary compensation in accordance with Prolific's standard practices. The study protocol was reviewed and approved by an institutional ethics committee.

To determine the required sample size, we conducted an *a priori* power analysis using G^*^Power (Faul et al., 2009). Assuming a small-to-medium effect (*f*^2^ = 0.10; Cohen, 2013) in a multiple-regression model, the analysis (alpha = 0.05, power = 0.80) indicated a minimum sample of 118. Because indirect effects are harder to detect, simulation evidence suggests bias-corrected bootstrap tests require samples in the low hundreds (Schoemann et al., 2017). The final sample (*N* = 353) exceeded these benchmarks.

The final sample consisted of 353 respondents occupying managerial positions (see Table 1). Respondents varied in gender (53% men), age (M = 37.9, SD = 11.6), education level (34% Bachelor's degree, 49% Master's degree), and family status (37% single, 42% married). Respondents represented diverse industry sectors, including IT and technology (21%), manufacturing (18%), financial services (16%), professional services (14%), retail and trade (13%), and healthcare and other sectors (18%). All respondents reported having worked with at least one freelancer in the past 12 months, ensuring responses reflected direct managerial experience. On average, respondents worked with freelancers at least once per quarter, and 62% reported personally considering a freelance career at some point. To ensure genuine influence over freelancer compensation, the Prolific screening required respondents to confirm direct or advisory authority over freelancer pay/budgets before being invited to the survey. Table 1 summarizes respondents' managerial roles and freelancer experience; 79.6% worked with freelancers at least quarterly, indicating routine, first-hand exposure to pay decisions.

**Table 1:** Descriptive statistics and sample characteristics (*N* = 353).

Variable	Category (dummy-code)	*N* (%)
Gender	Male (1)	187 (53.0)
	Female (2)	166 (47.0)
Age	Mean (SD)	37.9 (11.6)
	18-30 years (1)	112 (31.7)
	31-45 years (2)	158 (44.8)
	46 + years (3)	83 (23.5)
Education	Bachelor's degree (1)	120 (34.0)
	Master's degree (2)	173 (49.0)
	Other (3)	60 (17.0)
Family Status	Single (1)	131 (37.1)
	Married/partnered (2)	148 (41.9)
	Other (3)	74 (21.0)
Industry Sector	IT and technology (1)	74 (21.0)
	Manufacturing (2)	64 (18.1)
	Financial services (3)	56 (15.9)
	Professional services (4)	49 (13.9)
	Retail and trade (5)	46 (13.0)
	Healthcare and other (6)	64 (18.1)
Managerial role	CEO/General manager (1)	98 (27.8)
	Operations manager (2)	43 (12.2)
	Sales/Marketing manager (3)	76 (21.5)
	Administrative manager (4)	45 (12.7)
	Technical manager (5)	34 (9.6)
	CFO (6)	32 (9.1)
	HR manager (7)	25 (7.1)
Freelancer Exp.	Worked with freelancer (12 mo.) 1	353 (100.0)
	Works with freelancers regularly (quarterly+) (2)	281 (79.6)
	Personally considered freelance career (3)	219 (62.0)

Percentages may not sum to 100 due to rounding.

## Measures

### Freelancer Mindset Orientation (FMO)

Managers' freelancer-oriented career self-concept was captured using three indicators that reflect (a) openness to personally adopting freelance work, (b) general evaluation of freelancing as a labor-market trend, and (c) role-specific preferences for using freelancers. First, respondents reported their own intention to become a freelancer (“Do you think there is a reasonable possibility you might be a freelancer in the future?”) (1 = yes [62%], 2 = no [38%]). Second, they rated their evaluation of the freelancer trend on a 1 (very positive) to 5 (very negative) scale (M = 2.31, SD = 0.85). Because higher raw scores on these two indicators represented weaker rather than stronger freelancer orientation, both were reverse-coded before standardization so that higher scores consistently represented stronger FMO. Third, respondents indicated their preferred share of freelancers vs. salaried employees across six different roles. A role-based freelancer preference index was created by averaging responses across the six items (*M* = 1.84, SD = 0.39).

Because these indicators were assessed on different response scales, we treated FMO as a composite index based on z-standardized indicators averaged into an overall index (higher values = stronger FMO). We treat FMO as a composite index rather than a reflective scale because the three indicators capture conceptually distinct but theoretically related facets (personal career openness, attitudinal evaluation, behavioral preference), each measured on a different response format. This formative/composite approach is consistent with recommendations for multi-faceted attitudinal constructs where indicators are not interchangeable (Diamantopoulos and Winklhofer, 2001). To verify internal coherence, we confirmed that the three z-standardized components were positively and significantly intercorrelated, supporting their combination into a single index. In supplementary analyses, the three components were also examined separately. As each component was standardized before averaging, the composite has a mean of 0 by construction. A value of zero therefore represents the sample-average level of FMO rather than an absence of FMO, with positive and negative values indicating above and below-average FMO, respectively. The final composite was subsequently standardized, yielding the M = 0.00 and SD = 1.00 reported in Table 2.

**Table 2:** Means, standard deviations, and correlations among study variables.

Variable	M	SD	1	2	3	4	5	6	7	8
1. Gender	0.53	0.50								
2. Age	37.9	11.6	0.08							
3. Education	2.1	0.73	0.05	0.12^*^						
4. Sector	4.04	1.66	−0.16^**^	0.03	0.01					
5. Managerial role	3.46	1.98	0.14^*^	0.15^**^	0.08	0.21^**^				
6. FMO^a^	0.00	1.00	−0.03	0.09	0.07	−0.01	−0.04			
7. Inclusion	2.07	0.74	0.06	0.10	0.04	−0.08^*^	0.04	0.38^**^		
8. Evaluations	2.42	0.87	0.04	0.15^**^	0.06	0.01	0.04	0.29^**^	0.34^**^	
9. Pay equality	4.28	0.69	−0.13^*^	0.08	0.11^*^	−0.02	−0.05	0.25^**^	0.26^**^	0.16^**^

*N* = 353. FMO, Freelancer Mindset Orientation. ^a^standirzised score. ^*^p < 0.05. ^**^p < 0.01.

### Inclusion practices

Respondents reported whether freelancers, relative to salaried employees, participate in eight organizational practices covering HR-related processes (performance management, training), material inclusion (access to benefits), social inclusion (social events, organizational values), and strategic inclusion (strategic issues, organizational data). Responses were coded to capture the degree to which freelancers were treated equivalently to salaried employees: 1 = freelancers do not participate while employees do (exclusion), 2 = practices vary across freelancers (partial inclusion), 3 = no difference between freelancers and salaried employees (full inclusion). We note that response option 1 captures the case where freelancers are excluded from a practice that employees access; this is theoretically distinct from the case where neither group participates, which is excluded by design since all items refer to practices that exist in the organization. The inclusion index was computed as the mean of the 8 items (M = 2.07; SD = 0.74, alpha = 0.91) and standardized.

### Evaluations of freelancers

Managers' evaluations of freelancers were measured with five comparative items assessing freelancers relative to salaried employees on: (a) fit with organizational culture, (b) congruence with organizational values, (c) commitment, (d) loyalty, and (e) trustworthiness. Responses were on a 5-point scale from “freelancers much less than employees” to “freelancers much more than employees.” The comparative framing was chosen intentionally: it measures the manager's relational evaluation as a comparative judgment, which is the psychologically relevant signal for pay decisions. Managers set pay for freelancers not in absolute terms but relative to the employee benchmark. This framing does not imply employees are poorly matched; it captures variation in how favorably managers perceive freelancers relative to their internal reference point. An evaluation index was computed as the mean of its 5 items (*M* = 2.42; SD = 0.87, alpha = 0.86) and standardized.

### Pay equality

Managers' pay policies toward freelancers were assessed using six role-specific items: (1) a managerial role, (2) a core professional/expert role, (3) a non-core professional role, (4) a critical project role, (5) a routine operations role, and (6) a personalized occupation-of-choice scenario. For each role, respondents indicated what level of freelancer pay would be justified relative to a salaried employee in the same job. Thus, the dependent variable captures relative pay parity rather than absolute compensation, holding the occupational comparison constant within each scenario and thereby reducing the influence of absolute wage differences across jobs. An overall parity index was created by averaging the six roles (*M* = 4.28; SD = 0.69, alpha = 0.88).

### Control variables

We controlled for three demographic variables that prior research links to managerial attitudes toward workforce diversity and pay decisions. First, gender was included because research shows that women tend to hold more egalitarian pay attitudes and may evaluate external workers more favorably (Shore et al., 2003). Second, age was included because older managers accumulate broader experience with diverse work arrangements and hold more positive attitudes toward non-standard employment forms (Ng and Feldman, 2010). Third, education level was included because higher education is associated with lower categorical bias toward non-standard workers and stronger endorsement of equity-based pay norms (Chatard and Selimbegovic, 2007). Because our theoretical argument is that freelancer pay decisions reflect more than purely market-based considerations, we also sought to account for observable contextual factors that may capture variation in market-based determinants of compensation. We therefore controlled for industry sector, given well-established evidence of substantial inter-industry wage differentials even among comparably skilled workers (Krueger and Summers, 1988), and for respondents' managerial role, which may capture variation in managerial discretion and involvement in compensation-related decisions (Wangrow et al., 2015). Industry sector and managerial role were modeled as categorical variables using dummy indicators, with one reference category omitted for each set of indicators.

These controls nevertheless cannot capture all market-transaction characteristics relevant to freelancer compensation, such as freelancer-specific bargaining power, local wage benchmarks, real-time labor scarcity, or reputation. We therefore interpret the additional controls as accounting for observable contextual heterogeneity rather than as fully isolating all market forces and address this limitation explicitly below.

### Common method bias test

Because all constructs were reported by the same managers in a single, self-administered survey, common method variance (CMV) is a legitimate concern (Podsakoff et al., 2003). We addressed this concern with three complementary strategies rather than relying on a single diagnostic. First, we applied procedural (ex-ante) corrects: FMO, evaluations, inclusion, and pay-equality items were separated into distinct survey blocks, used different response formats and anchors (dichotomous, 5-point comparative, and role-based allocation scales), and were introduced with different framing and instructions, reducing the likelihood that respondents could infer or enact a consistent response pattern across constructs. Second, we conducted Harman's single-factor test (Chang et al., 2019); the unrotated first factor explained 20.69% of total variance, well below the 50% heuristic threshold. Third, as an additional diagnostic, we estimated a confirmatory single-factor model in which all indicators loaded on a single latent factor and compared its fit with that of the hypothesized multi-factor measurement model. The single-factor model demonstrated significantly poorer fit [χ^2^(30) = 158.17, *p* < 0.001, RMSEA = 0.09, CFI = 0.88, and TLI = 0.84], than the hypothesized multi-factor model [χ^2^(27) = 101.77, *p* < 0.001, RMSEA = 0.06, CFI = 0.95, and TLI = 0.92, Δχ^2^(3) = 56.40, *p* < 0.001], indicating that the observed covariance structure is not adequately represented by a single common factor. We also examined the pattern of zero-order correlations reported in Table 2. Several theoretically unrelated pairs of variables were not significantly correlated (e.g., gender with FMO, r = −0.03; education with FMO, r = 0.07), which is inconsistent with a pattern in which common method variance systematically inflates correlations across variables (Podsakoff et al., 2003). Taken together, these procedural and statistical checks suggest that common method variance is unlikely to fully account for the observed pattern of relationships, although it cannot be entirely ruled out given the cross-sectional, single-source design.

### Data analysis

Data were analyzed using Mplus 9 (Muthen and Muthen, 2010). We specified a path-analytic model in which FMO predicted managers' relational evaluations, which in turn predicted pay outcomes, while accounting for the direct path from FMO to pay. All models included gender, age, education, industry-sector and managerial-role dummy variables as controls. The indirect effect was estimated with bias-corrected bootstrapped confidence intervals based on 5,000 resamples (Hayes and Preacher, 2013; Schoemann et al., 2017). To test H5-H7, interaction terms were estimated for FMO × Inclusion and Evaluations × Inclusion. The FMO × Inclusion interaction was examined both in predicting pay equality (H5) and relational evaluations (H7), whereas the Evaluations × Inclusion interaction was examined in predicting pay equality (H6). Significant interactions were probed using conditional effects at one standard deviation above and below the mean of inclusion (Aiken et al., 1991).

## Results

Means, standard deviations, and correlations are presented in Table 2.

### Tests of hypotheses

The first regression analysis results (see Table 3, Model 1) indicate a positive association of Freelancer Mindset Orientation (FMO) on pay equality (β = 0.25^**^), supporting Hypothesis 1. Additionally, women presented higher levels of pay equality (M = 4.33, SD = 0.62) compared to men (M = 4.21, SD = 0.56).

**Table 3:** Regression results: predicting pay equality—direct effects, mediation, and moderation (H1, H3-H6).

Variable	Model 1 β	Model 2 β	Model 3 β	Model 4 β
Controls				
Gender	−0.13^*^	−0.12^*^	−0.11^*^	−0.11^*^
Age	0.08	0.06	0.05	0.05
Education	0.11^*^	0.10	0.10	0.10
Sector	−0.02	−0.02	−0.02	−0.01
Managerial role	−0.05	−0.05	−0.04	−0.04
Predictors				
FMO	0.25^**^	0.22^**^	0.21^**^	0.20^**^
Evaluations		0.16^*^	0.15^*^	0.15^*^
Inclusion			0.14^*^	0.13^*^
Interactions				
FMO × Inclusion				0.26^**^
Eval × Inclusion				0.03
R^2^	0.08^**^	0.11^**^	0.13^**^	0.19^**^
ΔR^2^ (interactions)				0.06^**^

*N* = 353. Standardized β coefficients reported. Model 1 = Controls and FMO only; Model 2 = adds Evaluations; Model 3 = adds Inclusion; Model 4 = adds interaction terms. Indirect effect (H4): β = 0.05, 95% CI [0.01, 0.09], based on 5,000 bootstrap resamples. ^*^p < 0.05. ^**^p < 0.01.

Hypothesis 2 suggested that FMO will be related to managers' evaluations of freelancers. Results (see Table 4, Model 2) confirm the hypothesis (β = 0.29^**^). Age was also related to evaluations such that an increase in age increases positive evaluations toward freelancers.

**Table 4:** Regression results: predicting evaluations of freelancers (H2 and H7).

Variable	Model 1 β	Model 2 β	Model 3 β	Model 4 β
Controls				
Gender	0.03	0.03	0.03	0.02
Age	0.15^**^	0.14^**^	0.14^**^	0.14^**^
Education	0.05	0.04	0.04	0.04
Sector	0.01	0.01	0.01	0.01
Managerial role	0.04	0.04	0.03	0.03
Predictors				
FMO		0.29^**^	0.28^**^	0.26^**^
Inclusion			0.33^**^	0.30^**^
Interactions				
FMO × Inclusion				0.24^**^
R^2^	0.03	0.08	0.11	0.15
ΔR^2^		0.05	0.03	0.04

*N* = 353. Standardized β coefficients reported. Model 1 = Controls only; Model 2 = adds predictor FMO (H2); Model 3 = adds Inclusion; Model 4 = adds interaction terms (H7).

^**^p < 0.01.

Consistent with Hypothesis 3, results (see Table 3, Model 2) indicated a significant positive relationship between evaluations and pay (β = 0.16^*^).

Hypothesis 4 proposed that the FMO-pay equality relationship would be mediated by managers' evaluations of freelancers. We tested this using the Mplus 9 model indirect command with bias-corrected bootstrapped confidence intervals (5,000 resamples). Consistent with the coefficients reported in Tables 3, 4, the standardized path from FMO to evaluations was a′ = 0.29 (*p* < 0.01) and the standardized path from evaluations to pay equality was b′ = 0.16 (*p* < 0.05). The corresponding standardized indirect effect (a′ × b′) was β = 0.05, 95% CI [0.01, 0.09], which excludes zero and therefore provides support for the hypothesized indirect association. The direct relationship between FMO and pay equality remained significant (β = 0.20, *p* < 0.01) after relational evaluations were included, indicating partial rather than complete statistical mediation. Hypotheses 5 and 6 proposed moderation by inclusion. The results (see Table 3, Model 4) support an interaction effect of FMO and inclusion on pay equality (β = 0.26^**^), supporting Hypothesis 5. No significant interaction between evaluations and inclusion on pay equality was found (β = 0.03, n.s.), and Hypothesis 6 was not supported.

Finally, we tested whether inclusion also moderates the relationship between FMO and relational evaluations (H7). The FMO × Inclusion interaction on evaluations was significant (β = 0.24^**^, see Table 4, Model 4), supporting Hypothesis 7.

Following Aiken et al. (1991), we plotted the interaction between FMO and inclusion on pay equality and between FMO and inclusion on evaluations at conditional values one SD above and below the means (see Figures 2, 3). The relationship between FMO and pay equality is stronger at high levels of inclusion, and the relationship between FMO and evaluations is stronger at high levels of inclusion.

**Figure 2:**
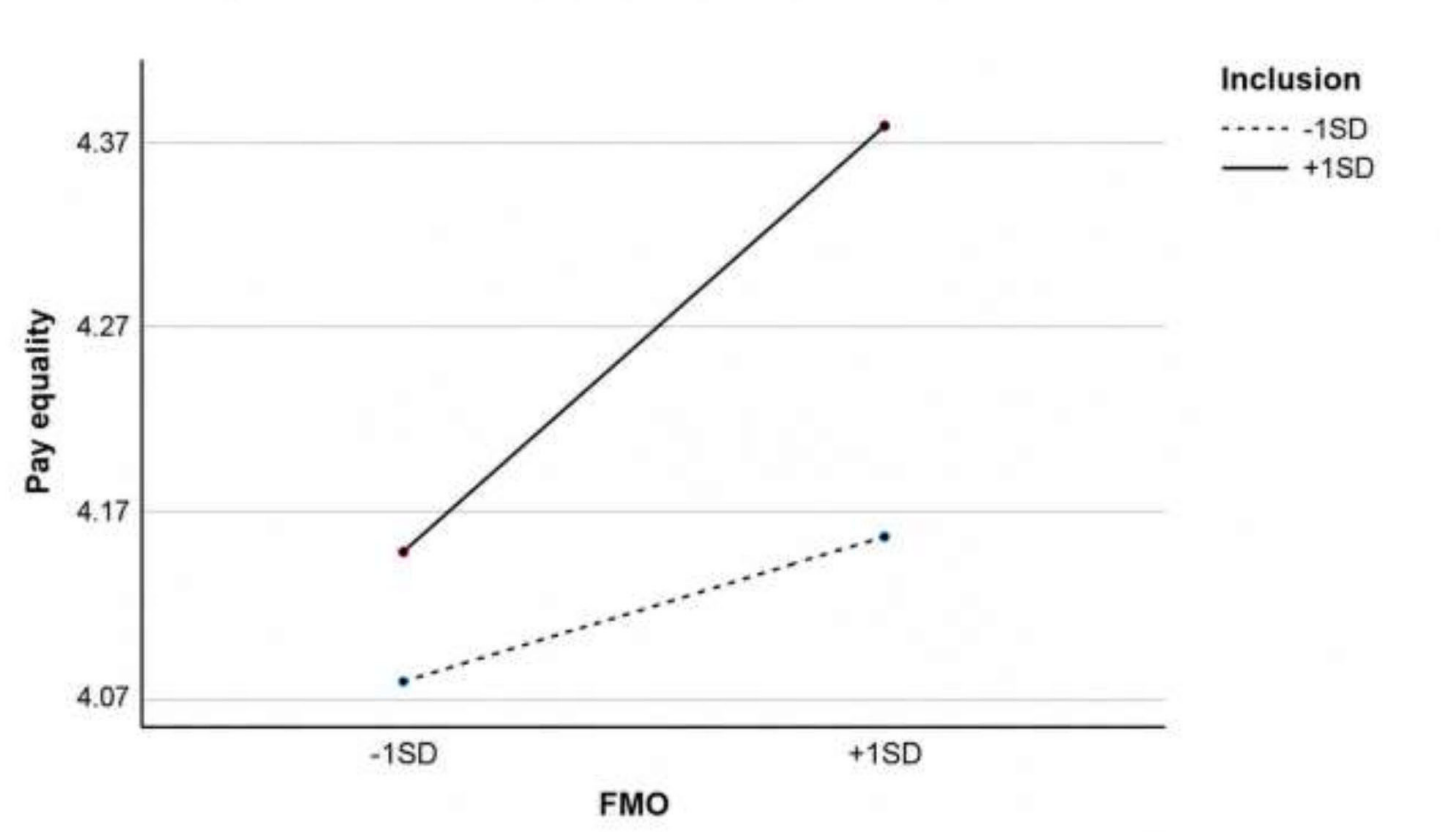
The relationship between FMO and pay equality as a function of inclusion level.

**Figure 3:**
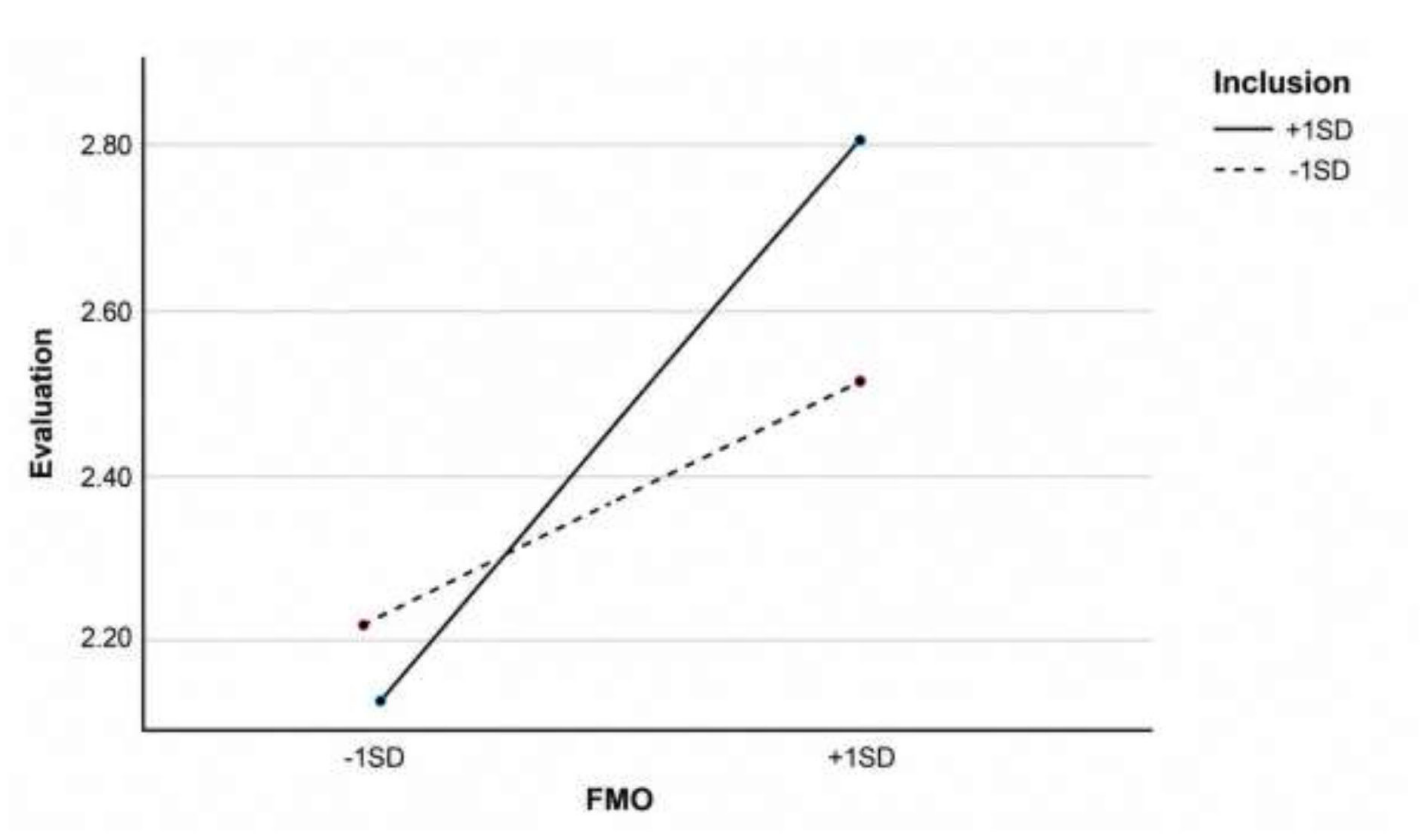
The relationship between FMO and relational evaluation as a function of inclusion level.

## Discussion

This study addresses a question that remains insufficiently explained by structural and institutional accounts of freelancer compensation: why do managers operating under broadly similar market, regulatory, and competitive conditions differ in the extent to which they support pay parity for freelancers performing comparable work? Although external constraints clearly shape compensation decisions, our findings suggest that part of this variation is also associated with how managers themselves understand and evaluate freelancing as a legitimate career form. Specifically, managers' Freelancer Mindset Orientation, defined as the extent to which they view freelancing as a legitimate, attractive, and personally resonant career mode, was positively associated with support for pay equality, both directly and indirectly through more favorable relational evaluations of freelancers.

### Theoretical contributions

The study advances research on freelancers and external workers by introducing and empirically testing FMO as a distinct, identity-based managerial orientation that shapes pay decisions. Prior work has emphasized macro- and meso-level determinants (Barley et al., 2017; Vallas and Schor, 2020; van den Groenendaal et al., 2023) while paying less attention to how individual managers' career self-concepts affect how they value freelancers. By bringing managerial cognition and identity into explanations of freelancer pay, the present study complements structural and institutional accounts with a micro-foundational perspective.

This contribution matters theoretically because it reframes freelancer pay inequality as a distributive outcome with a cognitive antecedent rather than a purely structural one. Institutional and labor-market accounts (Pfeffer, 1998; Vallas and Schor, 2020) explain why freelancers, as a category, tend to be discounted relative to salaried employees; they are less equipped to explain the substantial within-category variance that this study's data reveal, where individual managers facing ostensibly similar market and organizational constraints report markedly different pay-parity preferences. FMO supplies a plausible psychological explanation for that residual variance: it identifies a manager-level orientation in how legitimate freelancing is perceived to be, prior to and independent of any specific negotiation. This positions FMO not as a replacement for structural accounts of freelancer pay, but as the micro-level variable that may help explain how much of the available “structural room” for pay equity a given manager is willing to use. A related contribution lies in establishing relational evaluations as the mechanism through which identity-based orientations translate into resource allocation decisions. Managers higher in FMO evaluate freelancers as more fitting, loyal, and trustworthy, and these evaluations in turn predict higher pay equality, extending relational and psychological contract perspectives (Rousseau, 1995) by documenting a specific identity-infused pathway from managerial self-concept to concrete pay outcomes. This finding also has theoretical implications for distributive justice research, suggesting that fairness judgments toward contingent workers are not formed in a values vacuum but are shaped upstream by the perceiver's own career identity (Gerhart and Fang, 2014).

This finding also speaks to a long-standing tension in distributive justice research between allocation-rule accounts, which treat fairness judgments as the application of a norm (e.g., equity, equality, need) to a given case, and perceiver-centered accounts, which treat the norm itself as contingent on who is applying it (Gerhart and Fang, 2014). The present results favor the latter view for freelancer pay specifically: the same “equity” norm (pay commensurate with contribution) appears to be applied more or less generously depending on the manager's prior identity-based orientation toward the freelance category as a whole. This suggests that interventions aimed at improving distributive fairness for contingent workers cannot assume a shared, stable definition of “deserving” across managers; the definition itself is partly constructed by the manager's career-identity lens before any specific freelancer is even evaluated.

The study further enriches the emerging literature on inclusion in freelance work arrangements by positioning inclusion practices as a moderating condition that both relates directly to pay equality and conditions the relationship between FMO and pay equality. The finding that inclusion amplifies the FMO-pay link suggests that organizational context both contributes to pay-equality judgments and interacts with managerial orientations, consistent with person-situation interactionist models (Kristof-Brown et al., 2023). This connects inclusion perspectives with research on perceived insider status and organizational boundary-setting, showing that structural decisions about who belongs in organizational routines have downstream consequences for distributive outcomes (Ashforth and Mael, 1989; Stamper and Masterson, 2002).

The pattern of moderation findings further clarifies where inclusion appears to matter in the proposed process. Inclusion strengthened both the FMO-pay relationship and the relationship between FMO and relational evaluations, but it did not significantly strengthen the relationship between relational evaluations and pay. This pattern suggests that inclusion may operate primarily upstream, by creating the interactional and informational conditions through which a general orientation toward freelancing is translated into favorable judgments of particular freelancers and, ultimately, into compensation preferences. Once managers have formed target-specific judgments of fit, commitment, and trustworthiness, however, the relationship between those judgments and pay may be less dependent on the broader inclusion context. This interpretation helps explain why H6 was not supported while inclusion nevertheless plays a meaningful moderating role elsewhere in the model. Taken together, these contributions speak to a broader agenda in total talent management and pay equity research. The results show that organizations wishing to achieve more equitable treatment of external workers cannot rely solely on policy mandates or market rate benchmarks. The subjective layer of managerial interpretation, shaped by identity, relational judgments, and inclusion context, plays a significant and previously underestimated role in determining whether freelancers are rewarded as valued partners or discounted as peripheral vendors (Pfeffer, 1998; Shwartz-Asher et al., 2025).

More pointedly, the findings imply that pay equity for external workers is jointly produced by three forces operating at three different levels: individual identity (FMO), dyadic relationship quality (evaluations), and organizational structure (inclusion), and that interventions targeting only one level are likely to underperform. A policy mandate for pay parity imposed on a manager with low FMO and no inclusion infrastructure is unlikely to be enacted with the same fidelity as the identical mandate given to a manager who already regards freelancing as legitimate and who works within an inclusive structure; conversely, even a highly freelancer-oriented manager operating in an exclusionary organizational structure may lack the practical means to act on that orientation. This multi-level view reframes freelancer pay equity from a compliance problem into a person-organization alignment problem, which is a materially different design challenge for HR functions than the market-benchmarking approach that currently dominates practice.

### Practical implications

The findings carry several implications for organizations managing mixed workforces. Pay practices for freelancers are driven not only by market rates or central HR policies but also by managers' identities and relational interpretations. This means that interventions targeting managerial mindsets, such as leadership development programs, reflective workshops, or narrative-based exercises that expose managers to positive freelancer career stories, may shift FMO and, in turn, nudge pay decisions toward greater parity.

Because FMO's direct effect on pay equality persisted even after accounting for relational evaluations, mindset-focused interventions should not be assumed to work only by improving how managers perceive specific freelancers; part of their value likely lies in shifting managers' principled stance toward the freelance category as a whole, independent of any single working relationship. This argues for treating FMO development as a distinct HR lever alongside, not subordinate to, relationship-building interventions, and suggests that pay-equity training curricula should explicitly address category-level legitimacy beliefs (e.g., “is freelancing a real career?”) rather than only relationship-management skills.

Beyond mindset development, the mediating role of relational evaluations points to the value of structural conditions that allow trust-based relationships to form between managers and freelancers. Clear expectation-setting at the start of engagements, transparent performance criteria, and opportunities for repeated collaboration over multiple projects can strengthen perceptions of fit, commitment, and trustworthiness, shifting freelancers from the “transactional” to the “partner” category in managers' mental models (van den Groenendaal et al., 2023; Rousseau, 1995).

The moderating role of inclusion suggests that formal organizational practices and informal social signals matter in their own right. Ensuring that freelancers are invited to relevant meetings, integrated into communication channels, and given access to tools and strategic information creates a context in which favorable managerial orientations are more likely to translate into concrete pay decisions. HR functions can therefore treat inclusion design as a lever for pay equity, not only for belonging and engagement (van den Groenendaal et al., 2023; Nishii and Rich, 2013). The findings suggest that inclusion may matter in two ways: it is positively associated with pay equality in its own right and also strengthens the relationship between FMO and pay equality. Organizations may therefore benefit from combining efforts to foster more inclusive arrangements for freelancers with efforts to address managers‘ beliefs about the legitimacy and value of freelance careers. Rather than implying that either intervention is ineffective in isolation, the results suggest that their effects may be complementary. Future intervention research should test whether coordinated mindset- and inclusion-focused initiatives produce greater changes in actual compensation outcomes than either approach alone. A cross-cutting implication concerns the composition of managerial teams. The finding that women and older managers exhibited more equality-oriented pay attitudes and more positive evaluations of freelancers suggests that demographic diversity in management may have unintended yet positive consequences for pay equity toward external workers. Organizations that actively develop diverse management cohorts may therefore inadvertently foster a more freelancer-equitable compensation culture (Ng and Feldman, 2010; Shore et al., 2003). This implication should be stated cautiously: gender and age were included as statistical controls rather than theorized predictors, and their effects, while significant, were modest relative to FMO's. We therefore do not recommend that organizations treat management demographics as a substitute for direct intervention on FMO, relational evaluations, or inclusion; rather, this finding is best read as suggestive evidence that managerial diversity and freelancer pay equity may be positively linked through mechanisms this study was not designed to isolate, and which merit dedicated investigation before being used to justify diversity initiatives on pay-equity grounds alone.

### Limitations and future research

Several limitations warrant consideration. First, despite several efforts to address common method bias (see Method section), the cross-sectional, single-source design limits causal inference. Future research should use longitudinal, multi-wave, or experimental designs to establish temporal and causal ordering more clearly. Second, actual pay decisions are shaped by structural constraints, including market rates, labor shortages, compensation policies, and budget limitations, which may condition the influence of managers' orientations. Relatedly, the study relies on managers' self-reports rather than objective compensation data or freelancers' accounts. Future work should triangulate these reports with payroll records, platform data, or freelancer-reported rates to assess whether managerial preferences translate into realized pay outcomes (Guest and Conway, 2002; van den Groenendaal et al., 2023). Additionally, although participants reported either direct decision authority or formal input into freelancer compensation, we did not distinguish between final approval, budgetary authority, and advisory influence. Future research should examine whether FMO is more strongly related to realized pay among managers with greater compensation discretion. Furthermore, the Romanian context limits generalizability, as the relationships among FMO, relational evaluations, inclusion, and pay equality may vary across institutional environments with different employment protections, labor-market conditions, and norms regarding self-employment (Brewster et al., 2010; Vallas and Schor, 2020). Cross-national research is therefore needed to establish the model's scope conditions. Moreover, inclusion was treated as a unidimensional construct. Future research could distinguish formal inclusion, such as access to tools, benefits, and performance-management systems, from informal inclusion, such as social invitations and belonging cues. Finally, the model focuses on managers even though pay outcomes often emerge through negotiation. Freelancer bargaining behavior, reputation, outside options, career orientations, and market scarcity may shape whether FMO and relational evaluations translate into actual pay. Dyadic studies examining manager and freelancer characteristics jointly could therefore provide a more complete account of negotiated rates, relational evaluations, and relationship outcomes (Rousseau, 1995; van den Groenendaal et al., 2023). Although we added sector and managerial-role controls to proxy for market-based influences on freelancer pay, these proxies do not capture finer-grained market-transaction characteristics such as the freelancer's individual bargaining position, observed local market rates for the specific role, or real-time labor-market scarcity. Future research should incorporate direct market-rate benchmarks to more fully isolate FMO's association with pay equality from purely transactional determinants of freelancer pay.

### Conclusion

This study shows that managers with stronger Freelancer Mindset Orientation report greater support for pay equality between freelancers and comparable employees. Managers who view freelancing as a legitimate and attractive career mode also report more favorable evaluations of freelancers' fit, commitment, loyalty, and trustworthiness, and these relational evaluations partially account for the association between FMO and pay equality. Inclusion further strengthens the FMO-pay relationship and, the FMO-evaluations relationship, underscoring the role of organizational context in shaping when favorable managerial orientations are associated with compensation decisions. More broadly, this study argues that freelancer pay equity should be understood as the joint product of what a manager believes about freelancing as a category, how a manager judges the specific freelancers they work with, and how the organization structurally positions freelancers within its routines. Taken together, the findings point to complementary roles for managerial orientations, relational evaluations, and organizational inclusion in understanding freelancer pay equality. Future research should test whether interventions targeting these factors individually or jointly affect actual compensation decisions, ideally using longitudinal or experimental designs and freelancer-side data.

## Data Availability

The raw data supporting the conclusions of this article will be made available by the authors, without undue reservation.
